# *Mikania micrantha* Extract Inhibits HMG-CoA Reductase and ACAT2 and Ameliorates Hypercholesterolemia and Lipid Peroxidation in High Cholesterol-Fed Rats

**DOI:** 10.3390/nu12103077

**Published:** 2020-10-09

**Authors:** Azlinda Ibrahim, Nurul Husna Shafie, Norhaizan Mohd Esa, Siti Raihanah Shafie, Hasnah Bahari, Maizaton Atmadini Abdullah

**Affiliations:** 1 Department of Nutrition and Dietetics, Faculty of Medicine and Health Sciences, Universiti Putra Malaysia, Serdang 43400, Selangor, Malaysia; azlindaibrahim92@gmail.com (A.I.); nhaizan@upm.edu.my (N.M.E.); sitiraihanah@upm.edu.my (S.R.S.); 2 Laboratory of UPM-MAKNA Cancer Research, Institute of Bioscience, Universiti Putra Malaysia, Serdang 43400, Selangor, Malaysia; 3 Department of Human Anatomy, Faculty of Medicine and Health Sciences, Universiti Putra Malaysia, Serdang 43400, Selangor, Malaysia; haba@upm.edu.my; 4 Department of Pathology, Faculty of Medicine and Health Sciences, Universiti Putra Malaysia, Serdang 43400, Selangor, Malaysia; maizaton@upm.edu.my; 5 Institute of Bioscience, Universiti Putra Malaysia, Serdang 43400, Selangor, Malaysia

**Keywords:** *Mikania micrantha*, anti-hypercholesterolemia, lipid profile, steatosis, nutraceuticals

## Abstract

The present study aimed to determine the effect of an ethyl acetate extract of *Mikania micrantha* stems (EAMMS) in hypercholesterolemia-induced rats. Rats were divided into a normal group (NC) and hypercholesterolemia induced groups: hypercholesterolemia control group (PC), simvastatin group (SV) (10 mg/kg) and EAMMS extract groups at different dosages of 50, 100 and 200 mg/kg, respectively. Blood serum and tissues were collected for haematological, biochemical, histopathological, and enzyme analysis. Total cholesterol (TC), triglycerides (TG), low-density lipoprotein cholesterol (LDL-C), high-density lipoprotein cholesterol (HDL-C), aspartate aminotransferase (AST), alanine aminotransferase (ALT), urea, creatinine, malondialdehyde (MDA) level, as well as enzymes of HMG-CoA reductase (HMGCR) and acetyl-CoA acetyltransferase 2 (ACAT2), were measured. Feeding rats with high cholesterol diet for eight weeks resulted in a significantly (*p* < 0.05) increased of TC, TG, LDL-C, AST, ALT and MDA levels. Meanwhile, the administration of EAMMS extract (50, 100 and 200 mg/kg) and simvastatin (10 mg/kg) significantly reduced (*p* < 0.05) the levels of TC, TG, LDL-C and MDA compared to rats in the PC group. Furthermore, all EAMMS and SV-treated groups showed a higher HDL-C level compared to both NC and PC groups. No significant difference was found in the level of ALT, AST, urea and creatinine between the different dosages in EAMMS extracts. Treatment with EAMMS also exhibited the highest inhibition activity of enzyme HMGCR and ACAT2 as compared to the control group. From the histopathological examination, liver tissues in the PC group showed severe steatosis than those fed with EAMMS and normal diet. Treatment with EAMMS extract ameliorated and reduced the pathological changes in the liver. No morphological changes showed in the kidney structure of both control and treated groups. In conclusion, these findings demonstrated that EAMMS extract has anti-hypercholesterolemia properties and could be used as an alternative treatment for this disorder.

## 1. Introduction

Hypercholesterolemia is a metabolic disorder that mainly results in an elevated concentration of plasma low density lipoprotein (LDL) cholesterol [1]. Hypercholesterolemia has been associated with many cardiovascular diseases, including atherosclerosis, stroke, cerebral paralysis, myocardial infarction [2] and also inflammation and cancer [3]. One of the bigger challenges in modern medicine is the identification of a cure for hypercholesterolemia which does not confer side effects. In recent times, plant-sourced products have been considered to be possible novel therapeutic agents as these are considerably less toxic, cost-effective and most importantly, they produce no or relatively lesser side effects as compared to their synthetic counterparts.

*Mikania micrantha* Kunth originates from the tropical central and southern part of America and is extensively spread in the Pacific region and Southeast Asian countries. *M. micrantha* is traditionally used to treat stomach aches, jaundice, respiratory diseases, dysentery and rheumatism. This perennial creeping vine is also consumed as a juice as an alternative medicine for the treatment of diabetes, hypertension and hypercholesterolemia [4,5]. *M. micrantha* were previously reported to possess many health benefits such as antioxidant [6], anti-diabetic [7], anti-cancer [8,9], antiproliferative [10], anti-dermatophytic [11], anti-inflammatory [12] and antibacterial [5] activities. These beneficial effects are related to the richness of chemical constituents such as terpenoids, flavonoids, alkaloids and vitamins [5,6,11,13].

Despite its traditional use, scientific findings to prove the traditional claims of the anti-hypercholesterolemia properties of *M. micrantha* are limited. For that reason, this study aimed to determine the hypocholesterolemic potential of an ethyl acetate extract of *M. micrantha* stems (EAMMS) on male high-cholesterol-fed rats by determining the serum lipid profile [TC, total cholesterol; TG, triglycerides; HDL-C, high-density lipoproteins cholesterol; LDL-C, low-density lipoproteins cholesterol], lipid peroxidation, enzymatic activities and histopathological evaluation.

## 2. Materials and Methods

### 2.1. Reagents and Chemicals

Ethyl acetate (HmbG Chemical, Hamburg, Germany), ethanol (R&M Chemicals), haematoxylin (Sigma-Aldrich, St Louis, MO, USA), simvastatin (Pharmaniaga Logistics (M) Sdn. Bhd, Malaysia), 10% formalin (R&M Chemicals), xylene (R&M Chemicals) and eosin (Leica Biosystems Richmond Inc., Richmond, IL, USA) were used in this study. Pierce BCA Protein Assay Kits for protein quantification were purchased from Thermo Fisher Scientific (Rockford, IL, USA). Malondialdehyde (MDA) assay kits for lipid peroxidation assays were purchased from Elabscience Biotechnology Inc. (Houston, TX, USA). ELISA Kits (HMGCR and ACAT2) were purchased from Sunlong Biotech Co. Ltd. (Zhejiang, China). High cholesterol diet (1%) was purchased from EnvigoTeklad (Cambridgeshire, UK).

### 2.2. Sample Preparation 

*Mikania micrantha* was collected in August 2017 from Negeri Sembilan, Malaysia (GPS: 2.695652,102.160987) and a plant sample was deposited in the Forest Research Institute Malaysia (FRIM), Kepong, Selangor, Malaysia for taxonomic identification with a voucher specimen number of SBID 051/15. Fresh stems of *M. micrantha* was selected and washed and then dried at 35 °C for 72 h in a ventilated drying oven. *M. micrantha* powdered stems were then extracted using ethyl acetate and evaporated at 48 °C [6].

### 2.3. Animals

Sprague Dawley rats (male, 150–200 g weight) were purchased in this study. The rats were given tap water *ad libitum* and acclimatized under standardized laboratory circumstances (temperature 22 ± 2 °C; humidity 60 ± 4%; 12 h light-dark cycle). All animals used had received approval from the Universiti Putra Malaysia’s Institutional Animal Care and Use Committee (UPM/IACUC/AUP-R081/2017).

### 2.4. Animal Experimental Design

The rats were divided into six groups (*n* = 6). Group 1 as normal control (NC) was fed with a normal diet for 8 weeks. Group 2 to 6 were orally administered with high cholesterol diet (1%) throughout the study for 8 weeks. After the 4th week of the induction period, Group 2 was served as cholesterol-induced rats (PC). Group 3 was treated by orally administered via gavage with an aqueous suspension of simvastatin, SV (10 mg/kg) and groups of 4, 5 and 6 were orally administered via gavage with EAMMS at the dosage of 50, 100 and 200 mg/kg, respectively, during the treatment periods. The rats were treated for 4 weeks. Upon the administration of the last treatment dose, the animals were left to fast for 18 h. Blood samples were then acquired through the cardiac puncture approach with subjects under anaesthesia (ketamine/xylazine). Then, the blood samples were centrifuged at 3000 rpm at 4 °C for 10 min and the serum was stored at a temperature of −80 °C until the point of the assay. The liver and kidney were excised, weight and also stored at a temperature of −80 °C until the point of analysis.

### 2.5. Liver, Kidney and Haematogram Profile Analysis

Analysis of both liver and kidney profile including aspartate aminotransferase (AST), alanine aminotransferase (ALT), urea and creatinine were analyzed using a fully automated BiOLiS 24i Premium clinical analyzer (Hitachi, Bolton, UK). The total number of red blood cells (RBC), haemoglobin (Hb), packed cell volume (PCV), mean corpuscular volume (MCV), mean corpuscular haemoglobin volume (MCHC), white blood cell (WBC), icterus index, and plasma protein concentration were analysed.

### 2.6. Lipid Profile Analysis

The parameters that were analysed included total cholesterol (TC), triglycerides (TG), low density lipoproteins (LDL-C) and high density lipoproteins (HDL-C). The analyses were conducted using a fully automated clinical analyser (BiOLiS 24i Premium, Hitachi).

### 2.7. Serum Lipid Peroxidation

MDA level was quantified in the blood serum using a rat malondialdehydes (MDA) ELISA kit from Elabscience Biotechnology Inc., (Houston, TX, USA). All the procedures were conducted carefully according to the manufacturer’s instructions. The absorbance was measured at 450 nm.

### 2.8. Histopathological Examination

Briefly, 10% formalin buffer solution was used to fix the liver and kidney. Upon fixing, the tissues were subjected to paraffin embedding and being stained with haematoxylin and eosin (H&E) dye. The tissues of interest which have been stained were viewed and analysed using an image analyser. The histology of selected tissues was evaluated qualitatively and quantitatively. The microscopic analysis of all tissue samples was evaluated as a blind study by a pathologist and other researchers. Grading was conducted on the severity of steatosis which afflicted liver tissues and this was done according to the approach as described by Brunt et al. [14].

### 2.9. HMGCR and ACAT2 Activity Assays

Protein from liver tissue was homogenized in phosphate buffered saline (PBS, pH 7.4) at 4 °C. The supernatant was then centrifuged at 3500× *g* with 4 °C for 10 min and quantified with a BCA protein assay kit according to kit instructions. The levels of HMG-CoA reductase (HMGCR) and acetyl-CoA acetyltransferase 2 (ACAT2) enzymes were measured according to the ELISA protocol provided by the manufacturer.

### 2.10. Statistical Analysis

The data were expressed as mean ± standard error of the mean (SEM) for body and organ weight, haematological parameters, liver and kidney profile, lipid profile, lipid peroxidation and enzymatic analysis. All data were analysed using one-way analysis of variance (ANOVA) and Tukey’s multiple comparison tests (*p* < 0.05).

## 3. Results

### 3.1. Body and Organ Weights

**Figure 1:**
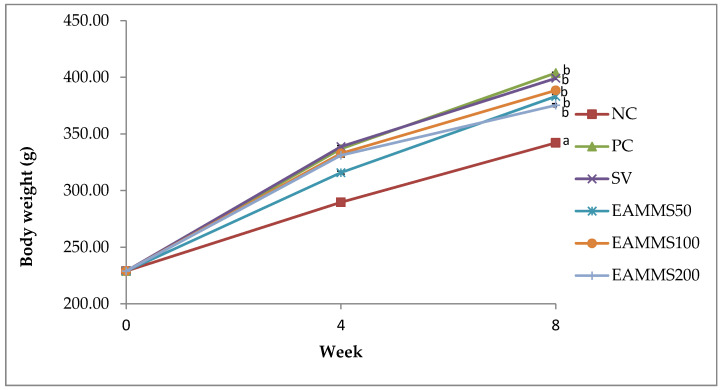
Effects of ethyl acetate extract of *Mikania micrantha* stems (EAMMS) on body weight in hypercholesterolemia rats. Values are expressed as mean ± SEM (*n* = 6). Means with different superscripts (a or b) indicate statistically different at *p* < 0.05 using Tukey’s multiple comparison test. NC—Normal control; PC—Positive control with high cholesterol diet (1%); SV—High cholesterol diet (1%) with simvastatin (10 mg/kg body weight); EAMMS50—High cholesterol diet (1%) with ethyl acetate *Mikania micrantha* stem extract (50 mg/kg body weight); EAMMS100—High cholesterol diet (1%) with ethyl acetate *Mikania micrantha* stem extract (100 mg/kg body weight); EAMMS200—High cholesterol diet (1%) with ethyl acetate *Mikania micrantha* stem extract (200 mg/kg body weight).

The changes in body weights of rats during the experiments are presented in Figure 1. All groups showed increased body weights throughout the study. The NC group exhibited an increasing body weight throughout the experimental period. After 4 weeks of induction with high cholesterol diet (HCD), the body weight of all HCD-induced groups was increased compared to the normal group (NC) but not significantly when compared to each other (Figure 1). Besides, there was a significant increased (*p* < 0.05) in the body weight of rats fed with high cholesterol diet for 8 weeks compared to the NC group. On the other hand, after the treatment period with EAMMS at different dosages, the body weight was shown to have lower body weight but not significant (*p* > 0.05) when compared to the PC group. Among the EAMMS-treated group, 100 mg/kg showed the highest body weight increment but not significant when compared to 50 mg/kg and 200 mg/kg treatment groups. The SV-treated group was showed no significant difference (*p* > 0.05) of body weight when compared to PC and EAMMS (50, 100 and 200 mg/kg) groups.

Table 1 indicates the weights of the livers and kidneys of the experimental rats. The liver weight from the PC group was significantly higher (*p* < 0.05) compared to the NC group and no difference with all EAMMS-treated groups. Meanwhile, there are no significant differences (*p* > 0.05) of kidney weight (right or left) between all groups.

### 3.2. Effect of EAMMS on Liver, Kidney and Haematogram Parameters

Table 2 shows the serum haematological, liver and kidney profiles of rats. Rats that were fed with a diet supplemented with 1% cholesterol for 8 weeks displayed a significant (*p* < 0.05) increase in AST, ALT and MCV levels. This is apparent when the PC group is compared against the NC group. Meanwhile, the levels of urea, creatinine, RBC, MCHC, WBC from the PC group showed slight increases but these were not significant (*p* > 0.05) when compared to the NC group. After 4 weeks of treatment, the levels of AST, ALT and MCV in the EAMMS-treated groups were not significant (*p* > 0.05) when compared to the PC group whilst the RBC, MCHC, WBC and additionally the kidney profiles (urea, creatinine), showed no significant differences between all groups. 

However, among the different dosages of EAMMS, 200 mg/kg resulted in a significant increase (*p* < 0.05) in the level of creatinine compared to the other dosages of EAMMS-treated and NC groups. In the SV-treated group, both AST and ALT levels showed significantly increases (*p* < 0.05) when compared to PC groups. Besides, there were no significant (*p* > 0.05) differences in Hb concentration and icterus index at all EAMMS dosages and controls. The PCV level also was not affected by the treatment of EAMMS in this study. For the leukocyte parameters, no significant differences were identified in terms of WBC count as well as differential leukocyte count.

### 3.3. Effect of EAMMS on Lipid Profile

Table 3 shows TC, TG, LDL-C and HDL-C levels in the serum of the experimental rats. The results show that feeding rats a diet supplemented with 1% cholesterol for 8 weeks resulted in a significant (*p* < 0.05) increase in TC, TG and LDL-C levels in the PC group compared to the NC group. After 4 weeks of the treatment (EAMMS and SV) period, there were significant (*p* < 0.05) decreases in serum TC, TG and LDL-C levels observed in the EAMMS-treated and SV groups compared to the PC group (Table 3). The levels of HDL-C in all EAMMS-treated groups and SV group showed no significant (*p* > 0.05) differences when compared to control groups (NC and PC). There were no significant differences (*p* > 0.05) in the level of serum TC, TG, LDL-C and HDL-C when compared between different dosages of EAMMS extract at week 8. Besides, the level of TC, TG, LDL-C and HDL-C in all EAMMS-treated groups were comparable and not significant (*p* > 0.05) as compared to the SV-treated group. 

### 3.4. Effect of EAMMS on Lipid Peroxidation

The levels of malondialdehyde (MDA) in the serum of experimental rats are listed in Table 4. Rats fed with a diet supplemented with 1% cholesterol over 8 weeks had significant (*p <* 0.05) increases in MDA levels when compared between the PC group and the NC group. After 4 weeks of the treatment period, there were marked and significant decreases (*p <* 0.05) in serum MDA levels observed in the EAMMS-treated and SV groups compared to the PC group but these were not significant when compared to the NC group. The comparison of different dosages of EAMMS extract did not show any significant differences (*p >* 0.05) in the serum MDA levels at week 8 (Table 4). 

### 3.5. Histopathological Results

**Figure 2:**
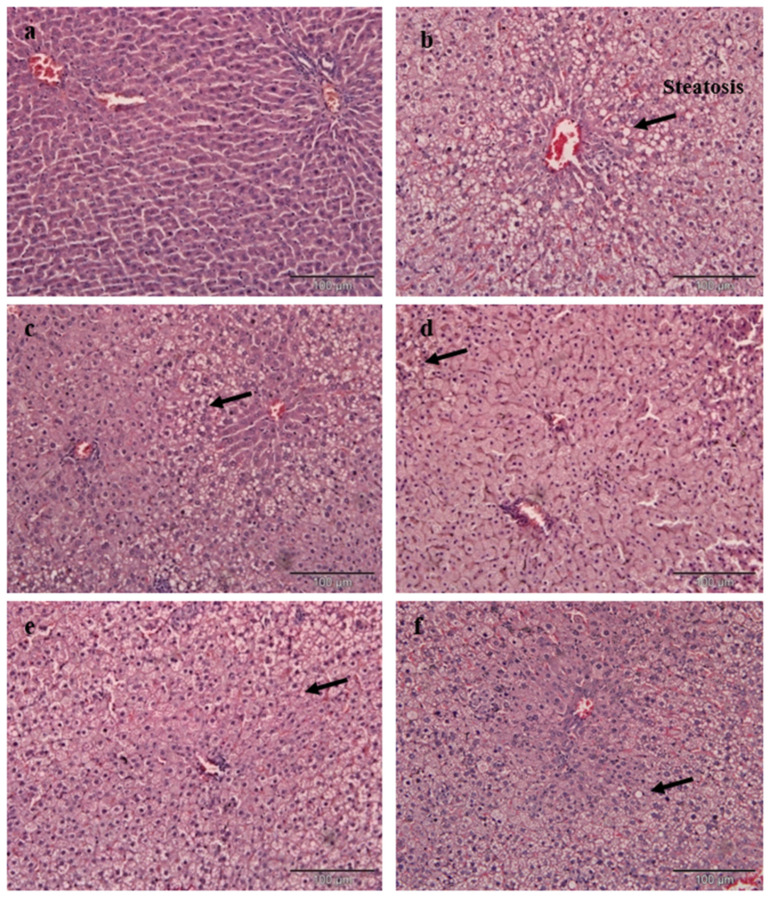
Histopathology of rat liver tissue in different groups. (**a**) NC—Normal control, (**b**) PC—Positive control with high cholesterol diet (1%), (**c**) SV—High cholesterol diet (1%) with simvastatin (10 mg/kg body weight), (**d**) EAMMS50—High cholesterol diet (1%) with ethyl acetate *Mikania micrantha* stem extract (50 mg/kg body weight), (**e**) EAMMS100 High cholesterol diet (1%) with ethyl acetate *Mikania micrantha* stem extract (100 mg/kg body weight), (**f**) EAMMS200—High cholesterol diet (1%) with ethyl acetate *Mikania micrantha* stem extract (200 mg/kg body weight). Livers were stained with hematoxylin and eosin (H&E) and visualized under a light microscope at 100× *g* magnification.

Table 5 shows the results for the liver histopathological scoring analysis of rats. Liver steatosis was absent in the NC group but observed in the PC group. There was a significant difference in steatosis between the PC group and those fed with EAMMS and NC diet. Liver tissues in the PC group showed severe diffuse steatosis within the hepatocytes and loss of single cell plates. The hepatocytes showed large cytoplasmic vacuoles due to fat deposition. However, there was no lobular or portal tract inflammation observed in the PC group. In the SV group, mild portal inflammation was observed accompanying moderate steatosis. There were expanded of losses single cell plates of hepatocytes with no significant lymphocytic infiltration in the portal tract. All EAMMS-treated liver had significantly less (*p* < 0.05) liver fat deposited than the PC group, with no inflammation, fibrosis or congestion on them that similar to that of the NC group (Table 5, Figure 2).

**Figure 3:**
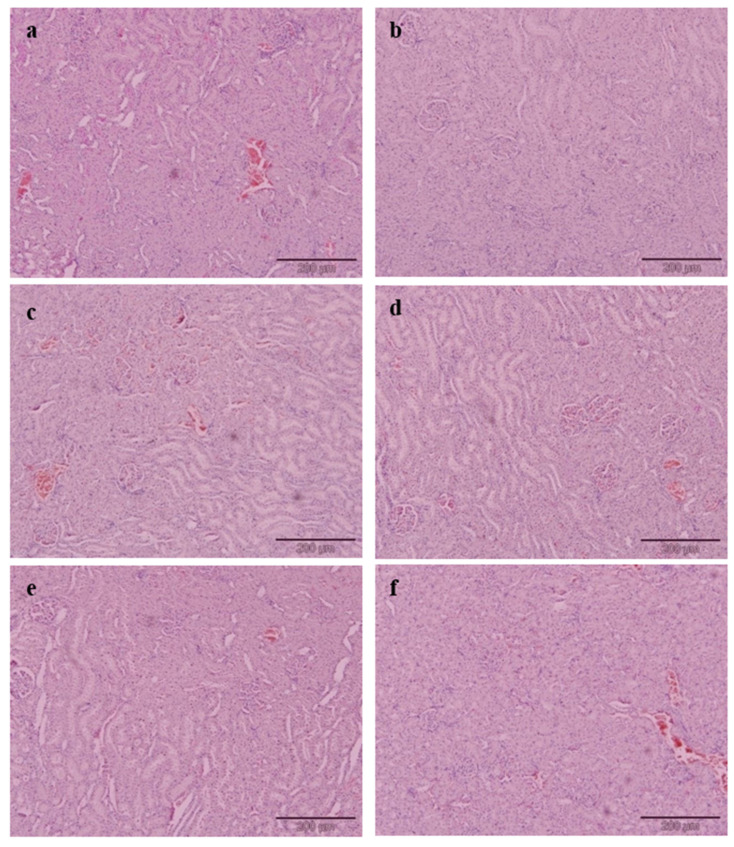
Histology of rat kidney tissue in different groups. (**a**) NC—Normal control, (**b**) PC—Positive control with high cholesterol diet (1%), (**c**) SV—High cholesterol diet (1%) with simvastatin (10 mg/kg body weight), (**d**) EAMMS50—High cholesterol diet (1%) with ethyl acetate *Mikania micrantha* stem extract (50 mg/kg body weight), (**e**) EAMMS100 High cholesterol diet (1%) with ethyl acetate *Mikania micrantha* stem extract (100 mg/kg body weight), (**f**) EAMMS200—High cholesterol diet (1%) with ethyl acetate *Mikania micrantha* stem extract (200 mg/kg body weight). Kidneys were stained with hematoxylin and eosin (H&E) and visualized under a light microscope at 200× *g* magnification.

The effects of EAMMS in kidney tissues are illustrated in Figure 3. None of the treated and control groups showed any morphological changes in kidney structure. The sections for each group showed normal appearance with regulated nuclear arrangement of uriniferous tubules and collecting tubules with normal looking glomeruli, there was absence of sclerosis, no mesangial proliferation and no inflammation in the kidney parenchyma.

### 3.6. Effect of EAMMS on HMGCR and ACAT2 Enzymes

The levels of HMG-CoA reductase (HMGCR) of experimental rats are given in Table 6. Rats that have been fed with a diet supplemented with 1% cholesterol over 8 weeks had significant (*p <* 0.05) increases in HMGCR levels when compared to the NC group. After 4 weeks of treatment, there were significant decreases (*p <* 0.05) in the HMGCR levels observed in the EAMMS-treated and SV groups compared to the PC group. Among the three different dosages of EAMMS extract, no significant difference (*p >* 0.05) was observed in the HMGCR levels at week 8 when compared to each other (Table 6).

Table 7 shows the levels of acetyl-CoA acetyltransferase 2 (ACAT2) in all experimental rats. Based on Table 7, rats that supplemented with 1% cholesterol over 8 weeks showed a significant (*p <* 0.05) increases in ACAT2 levels when compared to the NC group. After 4 weeks of treatment, rats in the EAMMS-treated and SV groups showed significantly decrease (*p <* 0.05) ACAT2 levels compared to the PC group. Among the three different dosages of EAMMS extract, the dosage of 50 mg/kg showed a slight decrease but no significant difference (*p >* 0.05) in the ACAT2 level compared to 100 mg/kg and 200 mg/kg at week 8. 

## 4. Discussion

Feeding with a cholesterol-enriched diet is one of the most commonly used methods for the induction of hypercholesterolemia in rats [15]. Administration of a high cholesterol diet to rats produces a marked increase of serum TC, TG and LDL-C as well as body weight when compared to a normal diet. A previous study revealed that a 1% increase in cholesterol intake by rats led to hypercholesterolemia, as evidenced by significant increases in serum TC, TG and LDL-C levels [16]. These observed changes are akin to those which could be expected when there is an excessive cholesterol load reaching the liver; a load that exceeds normal physiological limits. This will cause the inability of the liver to metabolize lipids thereby resulting in a relatively higher return of cholesterol into the blood circulation [17]. The relatively high levels of LDL which were found in rats which were fed with the cholesterol-enriched diet may be associated with the downward regulation of low density lipoprotein receptors (LDLR) by saturated fatty acids and dietary cholesterol [18]. According to Fungwe et al. [19], the elevation of triglycerides is caused by the cholesterol that was present in the diet which had been shown to diminish the oxidation of fatty acids and in the process, increase triglyceride and hepatic function levels.

Treatment with EAMMS extracts at all dosages showed marked decreases in serum TC, TG, and LDL-C levels and increases of HDL-C compared to the rats in the PC group after 4 weeks of supplementation. This phenomenon was explained by Adaramoye et al. [20] who reasoned that the decreased levels of cholesterol and triglycerides upon treatment are attributable to the lowering of the biosynthesis of hepatic triglycerides and the redeployment of cholesterol molecules between the molecules of lipoproteins. The effect of EAMMS extracts is also related to the abundance of chemical constituents’ presence in EAMMS extracts such as terpenoids, flavonoids, alkaloids and vitamins [5,6,11]. Terpenoids (particularly sesquiterpene lactones) are the major compounds found in the ethyl acetate extracts of the flowers, leaves and the whole part of *M. micrantha* [11]. The chemical profile of *M. micrantha* led to the identification of terpenoids such as stigmasterol, stigmasteryl-β-D-glucopyranoside, acetyl β-amyrin and lupeol [10]. The structural similarity of stigmasterol or plant sterol with cholesterol makes plant sterols some of the best substances in reducing cholesterol levels in the blood.

Several mechanisms have been postulated to clarify the process of cholesterol reduction by phytosterols or plant sterols. Plant sterols compete with biliary and also dietary cholesterol as an inhibitor to bind with mixed micelles for solubilization of micellar in the upper intestinal lumen that leads to the general reduction in the ability of the intestinal lining in absorbing cholesterol [21,22]. This hypothesis is consistent with the postulation byBrufau et al. [23] which showed that dietary plant sterols can diminish TC and LDL-C in animal and human models. Plant sterol and stanol ester increase LDLR mRNA and ex vivo LDLR protein expression in the monocytes as well as T-lymphocytes of humans and this changes correlated negatively with the changes of concentration of LDL in the blood, it may be postulated that upregulating LDLR expression leads to decreased LDL formation along the apolipoprotein B cascade [24].

One of the more important methods for diagnosing the cause of disease and the health status of rats is the assessment of haematological parameters. In this study, no significant changes were detected in all haematological values excluding MCV between EAMMS extract, simvastatin and the control groups. However, the administration of the EAMMS caused mild microcytic anemia due to the level of MCV in rat’s blood. MCV represents the average volume of the red blood cells. This abnormal blood condition could be caused by the presence of chemical constituents in plant extracts such as flavonoids and alkaloids saponins. Alkaloids have been shown to cause liver cirrhosis, liver megalocytosis and nodular hyperplasia [25] while terpenoids increase membrane permeability to divalent and monovalent ions [25].

Modern toxicology often involves the utilisation of blood sera or tissues as markers to assess damage to organs and cells besides the induction, activation and inhibition of enzymes. The kidneys and liver are organs which play major roles in the detoxification of metabolic substances [26]. AST and ALT are markers which are normally used to detect and assess injuries onto hepatocytes. It is noted that both these markers are introduced into the bloodstream following incidences where cell damage or necrosis occurs [27]. From the findings of this study, PC rats exhibited significant increases in AST and ALT levels as compared to NC rats. A similar elevation of AST and ALT levels was observed in hypercholesterolemic rats by the findings of Souza et al. [28]. In the broader sense, hypercholesterolemia is often associated with toxicity due to heightened levels of liver enzymes as well as the peroxidation of lipids that produces a lot of free radicals in blood sera and tissues [29,30].

Rats which were treated with simvastatin recorded significant elevations in AST and ALT levels. It is noteworthy that prominent adverse effects associated with the administration of the statin group are asymptomatic increases in liver transaminases as well as myopathy [31]. According to Castro et al. [32], the frequency of liver enzymes increased in a small proportion of those taking statins (2.5%). On the contrary, rats which were supplemented with EAMMS showed no differences in AST and ALT levels as compared to those in the NC group. It can, therefore, be deduced that the EAMMS treatment may suppress the level of AST and ALT in the blood and possibly help in the healing of the hepatic tissue damage, suggesting that plant extract can stabilize the plasma membrane as well as cures the damage of hepatic tissues [33].

It has been determined that one of the vital mechanisms of cellular damage that are caused by free radicals such as reactive oxygen species (ROS) is lipid peroxidation. One of the products of the peroxidation of lipid is malondialdehyde (MDA) and this is used as an index to indicate oxygen-free radicals’ levels. MDA constitutes the main fraction of aldehydes that are produced upon the metabolism of lipid hydroperoxides and is extensively used to quantify and for the determination of lipid peroxidation. Elevation of serum MDA and TC in the hypercholesterolemic rats without any treatment suggested that the occurrence of lipid peroxidation eventually caused hypercholesterolemia [34]. According to Yokozawa et al. [35], decreases in lipid peroxidation could result in a reduction in the probability of hypercholesterolemia. In this present study, treatment with EAMMS extract was found to confer protection against lipid peroxidation in hypercholesterolemia-induced rats. EAMMS-treated rats were used to reduce MDA concentration nearly normal level compared to the rats with high cholesterol diet, therefore suggesting that EAMMS might possess an antioxidant activity owing to the presence of its phenol, alkane hydrocarbons, flavonoids and phytosterol [6], thus suggesting that EAMMS leads to beneficial response on oxidative stress in hypercholesterolemic rats. Many previous studies have shown that plant polyphenols, flavonoids, carotenoids, vitamins can effectively lower the level of TC, TG, LDL and MDA in hyper-cholesterolemic rodents [36,37,38,39]. The present results were following the findings of Musolino et al. [40,41] that demonstrated that bergamot polyphenolic fraction (BPF) prevented the alteration of lipid profile in hypercholesterolemic rats, counteracting oxidative stress markers and also ameliorate the dysregulation of the lipoprotein metabolism; suggesting that the richness of antioxidant properties may play an important role in improving dyslipidemia.

Cholesterol-enriched diets resulted in a remarkable change of liver histology in the PC group. A large accumulation of lipid droplets within hepatocytes of the liver is known as steatosis and has been observed microscopically. The histopathological result of liver tissue in this study is consistent with the postulation by Zheng et al. [42], who found the same hepatic architecture with the presence of large fat vacuoles in high cholesterol rats. The administration of EAMMS extracts at all dosages and simvastatin (10 mg/kg) ameliorated and reduced the hepatic lipid droplets in hepatocytes of the liver. No histological changes have been observed in the kidney tissues in all the experimental rats. These facts indicated that the EAMMS were able to inhibit the accumulation of fat in the liver due to some flavonoids in the plant extract which down-regulates the enzyme HMG-CoA reductase, the key enzyme in the process of cholesterol biosynthesis. The interaction of bioactive compounds in the plant extract with enzyme-substrate complex caused the changes of the active site of the enzyme, thus prevents the formation of cholesterol. The bioactive compounds in *M. micrantha* may help to suppress the HMGCR activity and reduce cholesterol biosynthesis in the mevalonate pathway [43]. In agreement, the previous study was done by Gliozzi et al. [44], who also reported that the flavonoids and phenolic compounds in BPF inhibit the endogenous biosynthesis of cholesterol that mediated by HMG-CoA reductase enzyme on the mechanistic action of glycosylated polyphenols (bruteridin and melitidin), which is bind to the catalytic site of HMG-CoA reductase as an endogenous HMG-CoA substrate and causing inhibition of cholesterol synthesis.

**Figure 4:**
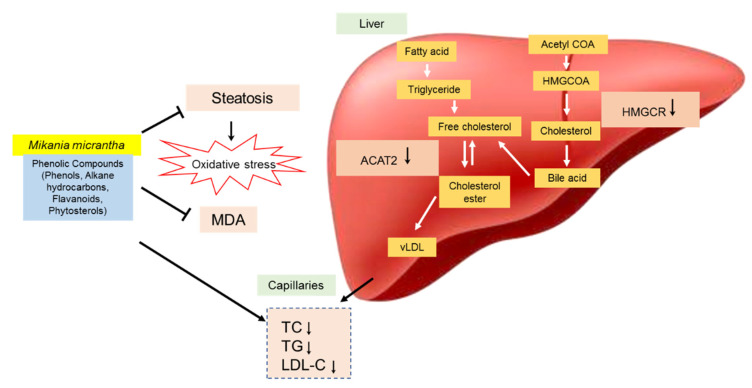
Proposed molecular mechanisms involved in hypocholesterolemic effects of *Mikania micrantha* stem extract (EAMMS) via inhibition of hepatic lipid accumulation, lipid peroxidation, HMG-CoA reductase (HMGCR) and ACAT2; leading to a decrease of cholesterol concentration.

The presence of caffeic acid ester, also known as chlorogenic acid such as 3,5-di-O-caffeoylquinic acid n-butyl ester in *M. micrantha* is reported [45,46]. The present findings suggested that these active compounds also accountable for the cholesterol-lowering activity, possibly mediated by down-regulation of HMGCR and up-regulation of LDL receptor in addition to down-regulating of ACAT2. Karthikesan et al. [47] also reported that the administration of chlorogenic acid for 45 days strongly reduced the activity of HMGCR and ACAT of lipid metabolism in rats. ACAT2 has important roles in cholesterol esterification, intestinal cholesterol absorption and ApoB-containing lipoprotein release [48]. It was reported that the inhibition of ACAT2 can effectively inhibit cholesterol absorption and reduce fat levels [49]. Phytosterols such as stigmasterol and sitosterol showed significantly reduced mRNA expression of ACAT2 activity in rodents [50]. The mechanism of the hypocholesterolemic action of *M. micrantha* extracts is summarized in Figure 4.

## 5. Conclusions

The present study showed that EAMMS extracts exhibited anti-hypercholesterolemia properties by improving lipid profile, enzyme inhibitory, reducing the lipid peroxidation and lipid accumulation in combating hypercholesterolemia.

## Figures and Tables

**Table 1:** Organ weight of rats at week 8.

Group	Liver	Kidney Right	Kidney Left
**NC**	9.06 ± 0.20 ^a^	1.12 ± 0.15 ^a^	1.16 ± 0.13 ^a^
**PC**	21.91 ± 0.39 ^b^	1.19 ± 0.11 ^a^	1.21 ± 0.05 ^a^
**SV**	20.57 ± 0.60 ^b^	1.22 ± 0.15 ^a^	1.24 ± 0.17 ^a^
**EAMMS50**	17.51 ± 0.83 ^b^	1.13 ± 0.13 ^a^	1.11 ± 0.19 ^a^
**EAMMS100**	18.40 ± 0.72 ^b^	1.18 ± 0.21 ^a^	1.15 ± 0.25 ^a^
**EAMMS200**	17.67 ± 0.45 ^b^	1.16 ± 0.16 ^a^	1.09 ± 0.15 ^a^

Values are expressed as mean ± SEM (*n* = 6). Means with different superscripts (a or b) in a column indicate statistically different at *p* < 0.05 by Tukey’s multiple comparison tests. NC—Normal control; PC—Positive control with high cholesterol diet (1%); SV—High cholesterol diet (1%) with simvastatin (10 mg/kg body weight); EAMMS50—High cholesterol diet (1%) with ethyl acetate *Mikania micrantha* stem extract (50 mg/kg body weight); EAMMS100—High cholesterol diet (1%) with ethyl acetate *Mikania micrantha* stem extract (100 mg/kg body weight); EAMMS200—High cholesterol diet (1%) with ethyl acetate *Mikania micrantha* stem extract (200 mg/kg body weight).

**Table 2:** Liver, kidney and haematology values of rats after treated with EAMMS extracts (week 8).

Parameters	NC	PC	SV	EAMMS 50	EAMMS 100	EAMMS 200
**AST (U/L)**	127.16 ± 1.78 ^a^	222.00 ± 6.06 ^b^	264.33 ± 4.72 ^c^	162.83 ± 7.36 ^a,b^	207.83 ± 3.07 ^a,b^	148.5 ± 4.01 ^a,b^
**ALT (U/L)**	71.00 ± 1.99 ^a^	226.00 ± 7.21 ^b^	191.00 ± 7.01 ^c^	134.00 ± 4.95 ^a,c^	194.00 ± 4.83 ^a,c^	182.00 ± 6.36 ^a,c^
**Urea (mmol/L)**	5.00 ± 0.30 ^a^	6.00 ± 0.39 ^a^	6.00 ± 0.21 ^a^	6.00 ± 0.46 ^a^	6.00 ± 0.26 ^a^	7.00 ± 0.48 ^a^
**Creatinine (umol/L)**	41.00 ± 1.10 ^a^	49.00 ± 0.80 ^a^	48.00 ± 0.86 ^a^	48.00 ± 1.34 ^a^	48.00 ± 1.07 ^a^	54.00 ± 1.11 ^b^
**RBC (10^12^/L)**	7.49 ± 0.20 ^a^	8.57 ± 0.29 ^a^	9.26 ± 0.25 ^a^	8.84 ± 0.10 ^a^	9.16 ± 0.16 ^a^	8.62 ± 0.17 ^a^
**Hb (g/L)**	149.50 ± 0.59 ^a^	144.83 ± 1.50 ^a^	155.33 ± 0.87 ^a^	152.33 ± 0.93 ^a^	159.17 ± 0.44 ^a^	149.67 ± 0.57 ^a^
**PCV (L/L)**	0.42 ± 0.04 ^a^	0.38 ± 0.07 ^a^	0.44 ± 0.05 ^a^	0.42 ± 0.03 ^a^	0.43 ± 0.02 ^a^	0.40 ± 0.03 ^a^
**MCV (Fl)**	55.90 ± 0.12 ^a^	44.15 ± 0.37 ^b^	47.59 ± 0.32 ^b^	47.20 ± 0.57 ^b^	46.48 ± 0.32 ^b^	46.26 ± 0.13 ^b^
**MCHC (g/L)**	358.55 ± 0.50 ^a^	382.61 ± 0.52 ^a^	353.25 ± 0.41 ^a^	365.41 ± 0.78 ^a^	374.56 ± 0.30 ^a^	375.86 ± 0.48 ^a^
**WBC (×10^9^/L)**	9.62 ± 0.62 ^a^	11.37 ± 1.13 ^a^	13.03 ± 0.44 ^a^	11.63 ± 0.73 ^a^	13.13 ± 0.62 ^a^	10.08 ± 0.68 ^a^
**Neutrophils (×10^9^/L)**	2.91 ± 0.36 ^a^	4.19 ± 1.23 ^a^	4.47 ± 0.78 ^a^	3.61 ± 0.57 ^a^	4.13 ± 0.45 ^a^	3.22 ± 0.90 ^a^
**Lymphocytes (×10^9^/L)**	6.12 ± 0.53 ^a^	6.34 ± 0.68 ^a^	7.66 ± 0.52 ^a^	7.15 ± 0.50 ^a^	8.08 ± 0.48 ^a^	6.26 ± 0.71 ^a^
**Monocytes (×10^9^/L)**	0.45 ± 0.18 ^a^	0.59 ± 0.36 ^a^	0.66 ± 0.19 ^a^	0.59 ± 0.28 ^a^	0.67 ± 0.22 ^a^	0.46 ± 0.21 ^a^
**Eosinophils (×10^9^/L)**	0.04 ± 0.30 ^a^	0.11 ± 0.17 ^a^	0.09 ± 0.23 ^a^	0.16 ± 0.31 ^a^	0.13 ± 0.20 ^a^	0.03 ± 0.29 ^a^
**Icterus index**	2.00 ± 0 ^a^	2.00 ± 0 ^a^	2.00 ± 0 ^a^	2.00 ± 0 ^a^	2.00 ± 0 ^a^	2.00 ± 0 ^a^
**Plasma protein (g/L)**	73.00 ± 0.49 ^a^	86.67 ± 0.68 ^b^	87.00 ± 0.33 ^b^	82.33 ± 0.51 ^b^	80.67 ± 0.44 ^b^	77.33 ± 0.40 ^b^

Values are expressed as mean ± SEM (*n* = 6). Means with different superscripts (a, b or c) in the same row indicate statistically different at *p* < 0.05 by Tukey’s multiple comparison test. NC—Normal control; PC—Positive control with high cholesterol diet (1%); SV—High cholesterol diet (1%) with simvastatin (10 mg/kg body weight); EAMMS50—High cholesterol diet (1%) with ethyl acetate *Mikania micrantha* stem extract (50 mg/kg body weight); EAMMS100—High cholesterol diet (1%) with ethyl acetate *Mikania micrantha* stem extract (100 mg/kg body weight); EAMMS200—High cholesterol diet (1%) with ethyl acetate *Mikania micrantha* stem extract (200 mg/kg body weight); AST—Aspartate aminotransferase; ALT—Alanine aminotransferase; RBC—Red blood cells; Hb—Haemoglobin; PCV—Packed cell volume; MCV—Mean corpuscular volume; MCHC—Mean corpuscular haemoglobin concentration; WBC—White blood cells.

**Table 3:** Serum lipid profiles of hypercholesterolemia-induced rats treated with different concentrations of *Mikania micrantha* stems (EAMMS) extracts at week 8.

Group	TC (mmol/L)	TG (mmol/L)	LDL-C(mmol/L)	HDL-C (mmol/L)
**NC**	1.18 ± 0.14 ^a^	0.56 ± 0.11 ^a^	0.24 ± 0.10 ^a^	1.27 ± 0.09 ^a^
**PC**	3.16 ± 0.39 ^b^	0.73 ± 0.17 ^b^	2.64 ± 0.39 ^b^	1.65 ± 0.36 ^a^
**SV**	1.45 ± 0.13 ^a^	0.41 ± 0.21 ^a^	1.09 ± 0.27 ^c^	1.59 ± 0.17 ^a^
**EAMMS50**	1.61 ± 0.18 ^a^	0.48 ± 0.14 ^a^	1.09 ± 0.28 ^c^	1.60 ± 0.25 ^a^
**EAMMS100**	1.53 ± 0.11 ^a^	0.46 ± 0.29 ^a^	1.12 ± 0.19 ^c^	1.61 ± 0.09 ^a^
**EAMMS200**	1.65 ± 0.38 ^a^	0.46 ± 0.33 ^a^	1.02 ± 0.26 ^c^	1.79 ± 0.55 ^a^

Values are expressed as mean ± SEM (*n* = 6). Means with different superscripts (a, b or c) in a column indicate statistically different at *p* < 0.05 by Tukey’s multiple comparison tests. NC—Normal control; PC—Positive control with high cholesterol diet (1%); SV—High cholesterol diet (1%) with simvastatin (10 mg/kg body weight); EAMMS50—High cholesterol diet (1%) with ethyl acetate *Mikania micrantha* stem extract (50 mg/kg body weight); EAMMS100—High cholesterol diet (1%) with ethyl acetate *Mikania micrantha* stem extract (100 mg/kg body weight); EAMMS200—High cholesterol diet (1%) with ethyl acetate *Mikania micrantha* stem extract (200 mg/kg body weight); TC: Total cholesterol; TG: Triglyceride; LDL-C: Low density lipoprotein cholesterol; HDL-C: High density lipoprotein cholesterol.

**Table 4:** Effect of ethyl acetate extract of *Mikania micrantha* stems (EAMMS) on lipid peroxidation using TBARS assay in hypercholesterolemia rats at week 8.

Group	MDA Level (ng/mL)
**NC**	137.71 ± 1.98 ^a^
**PC**	247.83 ± 5.98 ^b^
**SV**	142.06 ± 5.47 ^a^
**EAMMS50**	157.19 ± 3.93 ^a^
**EAMMS100**	143.54 ± 2.20 ^a^
**EAMMS200**	152.66 ± 3.68 ^a^

Values are expressed as mean ± SEM (*n* = 6). Means with different superscripts (a or b) indicate statistically different at *p* < 0.05 by Tukey’s multiple comparison tests. NC—Normal control; PC—Positive control with high cholesterol diet (1%); SV—High cholesterol diet (1%) with simvastatin (10 mg/kg body weight); EAMMS50—High cholesterol diet (1%) with ethyl acetate *Mikania micrantha* stem extract (50 mg/kg body weight); EAMMS100—High cholesterol diet (1%) with ethyl acetate *Mikania micrantha* stem extract (100 mg/kg body weight); EAMMS200—High cholesterol diet (1%) with ethyl acetate *Mikania micrantha* stem extract (200 mg/kg body weight); TBARS—Thiobarbituric acid reactive substances; MDA—Malondialdehyde.

**Table 5:** Liver steatosis and inflammation scores in rats of different groups.

Group	Steatosis	Inflammation
**NC**	-	-
**PC**	+++	-
**SV**	++	+
**EAMMS50**	++	-
**EAMMS100**	++	-
**EAMMS200**	+	-

Normal (-): No hepatocytes; Grade 1 (+): <33% of hepatocytes; Grade 2 (++): 33% to 66% of hepatocytes and Grade 3 (+++): >66% of hepatocytes involved [14].

**Table 6:** Effect of ethyl acetate extract of *Mikania micrantha* stems (EAMMS) on HMG-CoA reductase level in experimental rats at week 8.

Group	HMGCR (pg/mL)
**NC**	202.10 ± 6.68 ^a^
**PC**	271.50 ± 11.94 ^b^
**SV**	245.88 ± 15.94 ^c^
**EAMMS50**	258.54 ± 13.70 ^c^
**EAMMS100**	254.22 ± 8.64 ^c^
**EAMMS200**	255.33 ± 21.33 ^c^

Values are expressed as mean ± SEM (*n* = 6). Means with different superscripts (a, b or c) indicate statistically different at *p* < 0.05 by Tukey’s multiple comparison tests. NC—Normal control; PC—Positive control with high cholesterol diet (1%); SV—High cholesterol diet (1%) with simvastatin (10 mg/kg body weight); EAMMS50—High cholesterol diet (1%) with ethyl acetate *Mikania micrantha* stem extract (50 mg/kg body weight); EAMMS100—High cholesterol diet (1%) with ethyl acetate *Mikania micrantha* stem extract (100 mg/kg body weight); EAMMS200—High cholesterol diet (1%) with ethyl acetate *Mikania micrantha* stem extract (200 mg/kg body weight).

**Table 7:** Effect of ethyl acetate extract of *Mikania micrantha* stems (EAMMS) on ACAT2 levels in all treated experimental rats at week 8.

Group	ACAT2 (pg/mL)
**NC**	263.07 ± 1.01 ^a^
**PC**	529.46 ± 4.50 ^b^
**SV**	349.07 ± 2.40 ^c^
**EAMMS50**	405.87 ± 0.76 ^c^
**EAMMS100**	497.96 ± 1.33 ^c^
**EAMMS200**	454.42 ± 3.33 ^c^

Values are expressed as mean ± SEM (*n* = 6). Means with different superscripts (a, b or c) indicate statistically different at *p* < 0.05 by Tukey’s multiple comparison tests. NC—Normal control; PC—Positive control with high cholesterol diet (1%); SV—High cholesterol diet (1%) with simvastatin (10 mg/kg body weight); EAMMS50—High cholesterol diet (1%) with ethyl acetate *Mikania micrantha* stem extract (50 mg/kg body weight); EAMMS100—High cholesterol diet (1%) with ethyl acetate *Mikania micrantha* stem extract (100 mg/kg body weight); EAMMS200—High cholesterol diet (1%) with ethyl acetate *Mikania micrantha* stem extract (200 mg/kg body weight).
